# Real-world effectiveness of zanubrutinib plus salvage chemotherapy in relapsed or refractory double-expressor DLBCL

**DOI:** 10.1007/s00277-026-06974-6

**Published:** 2026-04-15

**Authors:** Jinbo Lu, Yujie Zhang, Xuan Ye, Jingru Shi, Qiuyan Lin, Ran Li, Xinyi Du, Lei Cao, Tian Tian, Jvjuan Wang, Yi Xia, Hailing Liu, Yuexin Cheng, Lei Fan, Haorui Shen, Sanmei Wang

**Affiliations:** 1 Department of Hematology, The First Affiliated Hospital of Nanjing Medical University, Jiangsu Province Hospital, Nanjing, 210000 China; 2 Department of Hematology, The First People’s Hospital of Yancheng, The Yancheng Clinical College of Xuzhou Medical University, Yancheng, 224000 China

**Keywords:** Zanubrutinib, BTK inhibitor, Double-expressor diffuse large B-cell lymphoma, Relapsed/refractory, Salvage therapy

## Abstract

To evaluate the efficacy and safety of the Bruton tyrosine kinase (BTK) inhibitor zanubrutinib in combination with salvage chemotherapy in patients with relapsed or refractory double-expressor diffuse large B-cell lymphoma (R/R DE-DLBCL). We performed a retrospective analysis of 35 patients with R/R DE-DLBCL treated at two hematology centers from June 2021 to June 2024. All patients received zanubrutinib combined with one of several salvage regimens, including zR-MINE, zR-DHAP, zR-GemOx, or zR2. Endpoints included objective response rate (ORR), complete response rate (CRR), progression-free survival (PFS), overall survival (OS), and adverse events (AEs). The median age was 60 years, and 91.4% of patients had the non-germinal center B-cell (non-GCB) subtype. At the end of therapy, the ORR and CRR were 45.7% (16/35) and 42.9% (15/35), respectively. After a median follow-up of 33.0 months, median PFS and OS were 8.0 and 12.0 months, respectively. Relapsed patients achieved a significantly higher CRR than refractory patients (56.5% vs. 16.7%, *p* = 0.034). On multivariate analysis, longer duration of response to prior therapy was an independent protective factor for OS (HR 0.88, 95% CI 0.77–0.99; *p* = 0.041). Grade 3–4 hematologic AEs were mainly neutropenia (42.8%) and thrombocytopenia (14.3%). Pulmonary infection was the most common non-hematologic AE (34.3%), and overall toxicity was manageable. Zanubrutinib combined with salvage chemotherapy demonstrates clinically meaningful activity with a manageable safety profile in patients with R/R DE-DLBCL and may represent a therapeutic option for this high-risk population. Sensitivity analysis confirmed the robustness of the observed survival benefit.

**Supplementary Information:**

The online version contains supplementary material available at 10.1007/s00277-026-06974-6.

## Introduction

Diffuse large B-cell lymphoma (DLBCL) is a biologically heterogeneous malignancy with particularly poor outcomes in the relapsed or refractory (R/R) setting. In the SCHOLAR-1 study, a large international retrospective analysis of 635 patients with R/R DLBCL, conventional salvage chemotherapy achieved an ORR of only 26% and a CRR of 7%, with a median overall survival (OS) of 6.3 months [1]. These limited outcomes leave many patients ineligible for autologous hematopoietic stem-cell transplantation (auto-HSCT). Even among those who undergo transplantation, the 3 year OS generally remains below 30% [2], highlighting a critical unmet clinical need.

Double-expressor lymphoma (DE-DLBCL), defined by concurrent overexpression of MYC and BCL-2 proteins, represents an aggressive DLBCL subtype characterized by chemoresistance and poor prognosis [3, 4]. Biologically, many DE-DLBCL tumors arise from post-germinal center B cells and depend on sustained B-cell receptor (BCR) signaling, providing a strong rationale for targeting Bruton’s tyrosine kinase (BTK) [5]. Although the first-generation BTK inhibitor ibrutinib is clinically approved, it has demonstrated only modest activity in R/R DLBCL, with an ORR of 23% [6], underscoring the need for more effective therapies. Zanubrutinib, a next-generation BTK inhibitor, offers improved selectivity and pharmacokinetics, resulting in enhanced efficacy and a favorable safety profile across B-cell malignancies [7]. By potently inhibiting BCR signaling, zanubrutinib may sensitize malignant B cells to chemotherapy.

However, real‑world evidence on the use of zanubrutinib combined with salvage chemotherapy in R/R DE‑DLBCL remains scarce. To bridge this knowledge gap, we conducted a retrospective analysis of R/R DE-DLBCL patients who were treated with this combination regimen. This study evaluated the antitumor activity, safety, and survival outcomes to inform clinical decision‑making for this high‑risk population.

## Materials and methods

### Patients

This retrospective study included patients with R/R DE-DLBCL treated at two hematology centers—the First Affiliated Hospital of Nanjing Medical University and First People’s Hospital of Yancheng—from June 2021 to June 2024. All patients received zanubrutinib in combination with salvage chemotherapy. Clinicopathologic characteristics and follow-up information were obtained from electronic medical records and supplemented by telephone interviews when needed.

Eligible patients were required to be ≥ 18 years of age, have histologically confirmed DE-DLBCL with immunohistochemical overexpression of MYC (≥ 40%) and BCL-2 (≥ 50%), and have relapsed or refractory disease after at least one prior line of therapy. Refractory disease was defined as progressive disease (PD) or stable disease (SD) as the best response to chemotherapy, or relapse within 12 months of the last treatment. Primary refractory disease was defined as failure to achieve complete or partial response to first-line therapy, or relapse within 3 months of response. Exclusion criteria included central nervous system involvement, double-hit or high grade B cell lymphoma, EBV-positive DLBCL, or prior exposure to BTK inhibitors.

Only patients with complete clinical and follow-up data were included in the final analysis. The final follow‑up date was August 31, 2025. The study was conducted in accordance with the principles of the Helsinki Declaration and was approved by the Institutional Ethics Committee of the First Affiliated Hospital of Nanjing Medical University (ethical approval: 2025-SR-121). Owing to the retrospective nature of the study, which involved only the collection and analysis of existing medical records, the requirement for written informed consent specifically for this research was waived by the ethics committee.

### Treatment

**Fig. 1:**
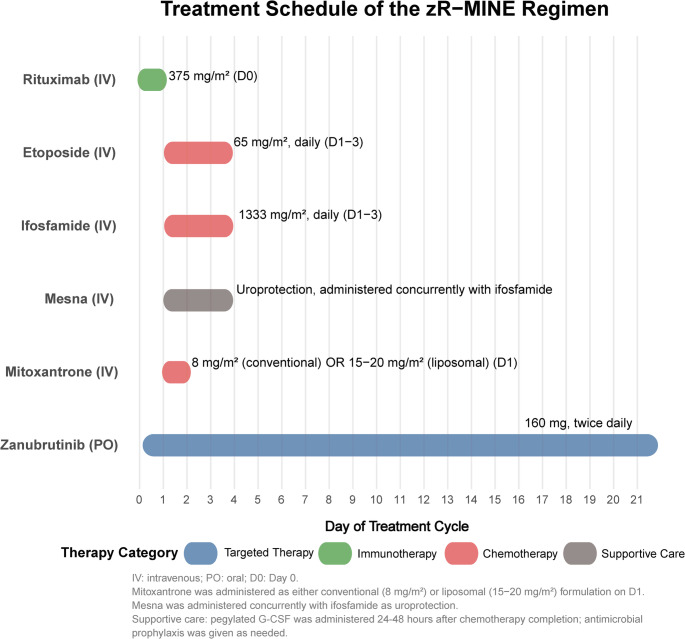
Treatment schedule of the zR-MINE regimen Zanubrutinib was administered orally twice daily throughout the 21-day cycle. Rituximab was infused on Day 0, while chemotherapy (ifosfamide, etoposide, and mitoxantrone) with concurrent mesna was given on Days 1–3. Mitoxantrone was administered as either conventional (8 mg/m²) or liposomal (15–20 mg/m²) formulation. Supportive care with pegylated G-CSF and antimicrobial prophylaxis was administered per protocol. Therapies are color-coded by category

All patients received zanubrutinib 160 mg orally twice daily, administered continuously in 21-day cycles, combined with an investigator-selected salvage regimen. The regimen choice was based on clinical judgment, considering the patient’s performance status, organ function, and prior toxicity, while avoiding previously administered therapies. Regimens included R-MINE (rituximab, ifosfamide, etoposide, mitoxantrone), R-ICE (rituximab, ifosfamide, carboplatin, etoposide), R-GDP (rituximab, gemcitabine, dexamethasone, cisplatin), R-DHAP (rituximab, dexamethasone, cytarabine, cisplatin), and R-GemOx (rituximab, oxaliplatin, gemcitabine). A non-chemotherapy regimen, R2 (rituximab plus lenalidomide), was also used. The number of cycles was up to 8 cycles with response. Doses of chemotherapy were reduced by 20% to 50% in the event of grade 4 adverse events. All patients received prophylactic pegylated granulocyte colony-stimulating factor (Peg-G-CSF). For patients who achieved a response and were eligible, autologous hematopoietic stem cell transplantation was recommended as consolidation therapy. The detailed schedule of a representative regimen (zR-MINE) is provided in Fig. 1. For other regimens, chemotherapy was administered on their designated days per standard schedules, while zanubrutinib was given continuously throughout each cycle, following the same principle.

### Study assessments

Treatment response was assessed according to the Lugano 2014 criteria, including complete response (CR), partial response (PR), stable disease, and progressive disease. Response evaluation included ^18F-FDG PET/CT at mid-treatment and at the end of induction, followed by contrast-enhanced CT every 3 months for 2 years. Follow up information was obtained from medical records and telephone interviews through August 31, 2025.

The primary endpoint was objective response rate (ORR), defined as the proportion of patients achieving CR or PR. Secondary endpoints included progression-free survival (PFS) and OS. PFS was defined as the time from initiation of combination therapy to disease progression, relapse, death from any cause, or last follow-up. OS was defined as the time from initiation of therapy to death from any cause or last follow up. Adverse events (AEs) were recorded and graded according to the National Cancer Institute Common Terminology Criteria for Adverse Events (CTCAE), version 5.0.

### Statistical analysis

Statistical analyses were performed with GraphPad Prism 10 and RStudio version 4.3.2. Continuous and categorical variables were presented as median (range) and frequency (percentage), respectively. Group comparisons used the χ² test or Fisher’s exact test, as appropriate. Survival was analyzed by the Kaplan-Meier method with log-rank test. Prognostic factors were assessed using univariate and multivariate Cox proportional hazards models; variables with *p* < 0.2 in univariate analysis were included in the multivariate model to mitigate overfitting. A sensitivity analysis was conducted to assess the robustness of overall survival, in which deaths not attributed to lymphoma progression were censored, and disease-specific survival was estimated. P values were two-sided, with *p* < 0.05 considered significant.

## Results

### Patients’ characteristics

A total of 35 patients with R/R DE-DLBCL treated with zanubrutinib plus salvage chemotherapy were included. The cohort comprised 18 men (51.4%) and 17 women (48.6%), with a median age of 60 years (range, 22–82 years); 51.4% (*n* = 18) were aged ≥ 60 years. The non GCB subtype predominated (91.4%, *n* = 32), and most patients (77.1%, *n* = 27) had Ann Arbor stage III-IV disease. High-risk features were frequent: 48.6% (*n* = 17) had an IPI score of 3–5, 62.9% (*n* = 22) had a Ki-67 index ≥ 80%, and 40.0% (*n* = 14) presented with B symptoms.

All patients had received at least one prior systemic therapy; 22 (62.9%) had relapsed disease and 13 (37.1%) had refractory disease. The median number of prior treatment lines was 1 (range, 1–3 lines), and 31.4% (*n* = 11) had received ≥ 2 lines. Most patients (94.3%, *n* = 33) had previously been treated with R-CHOP or an R-CHOP-like regimen. Four patients had undergone autologous stem-cell transplantation, and two had received CD19-directed CAR-T therapy.

### Efficacy

**Fig. 2:**
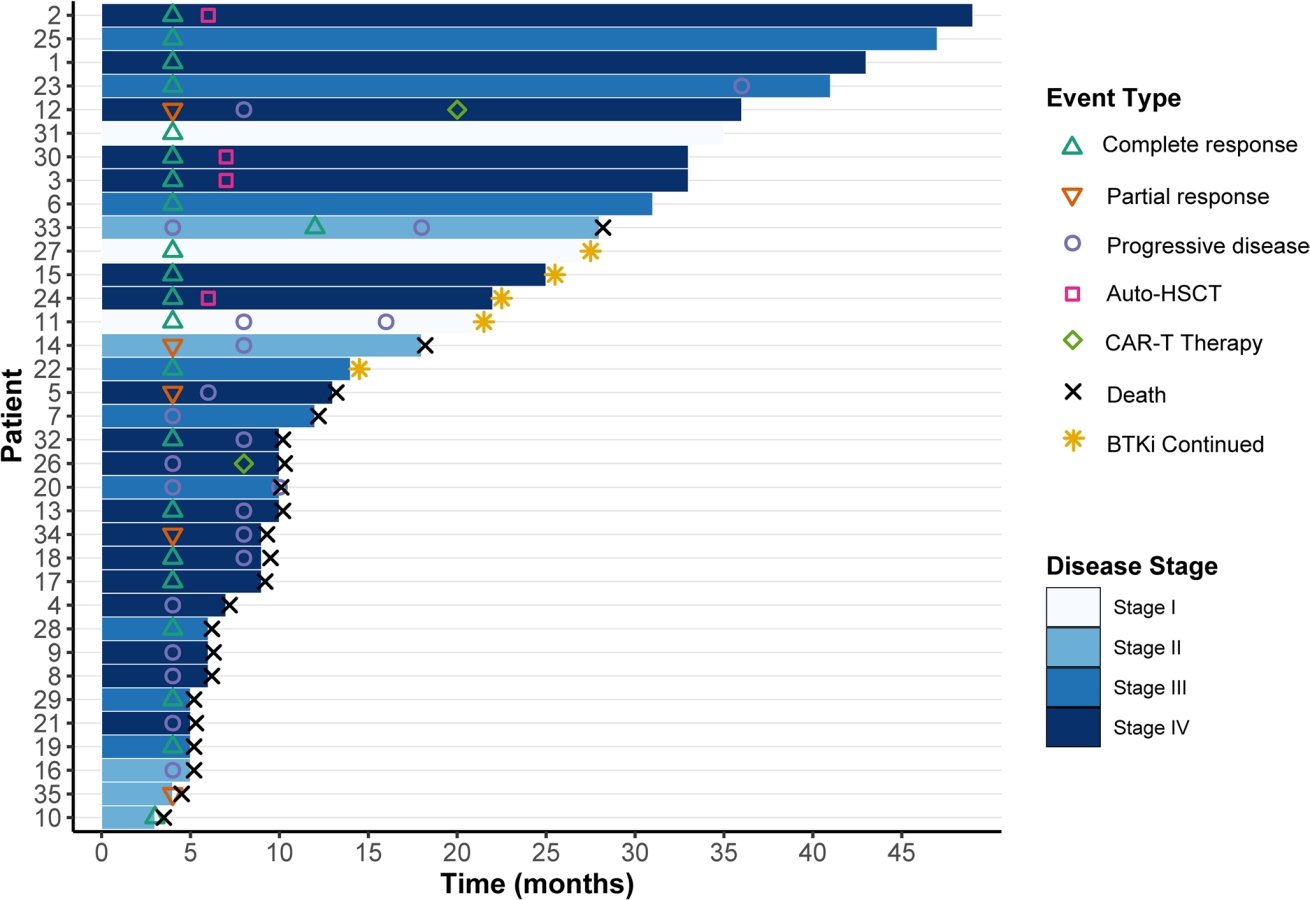
Treatment course and response dynamics in 35 patients with R/R DE-DLBCL receiving zanubrutinib plus salvage chemotherapy. Each horizontal bar represents an individual patient. The bar length corresponds to treatment duration. Bar color intensity represents the Ann Arbor stage at study enrollment, with darker shades indicating more advanced disease (Stage I-II: light blue; Stage III-IV: dark blue). Key clinical events are marked as follows: complete response (CR), partial response (PR), progressive disease (PD), autologous stem cell transplantation (Auto-HSCT), chimeric antigen receptor T-cell therapy (CAR-T therapy), death, and ongoing zanubrutinib treatment (BTKi continued)

Patients received one of several zanubrutinib-based regimens: zR-MINE (*n* = 8, 22.9%), zR-DHAP (*n* = 8, 22.9%), zR-GemOx (*n* = 6, 17.1%), zR-GDP (*n* = 2, 5.7%), zR-ICE (*n* = 2, 5.7%), or the non-chemotherapy oral regimen zR₂ (*n* = 9, 25.7%). Treatment courses are summarized in Fig. 2. The median number of chemotherapy cycles delivered for the entire cohort was 4 (range, 0–8 cycles). Notably, 10 patients (28.6%) required chemotherapy dose reduction, primarily due to treatment-related toxicities, with 7 of these patients (70%) being over 75 years of age. These dose adjustments, indicative of attenuated chemotherapy exposure in a subset of patients, precluded a formal calculation of relative dose intensity across the heterogeneous regimens. The median duration of zanubrutinib treatment was 8 months (range 3–32 months), reflecting treatment that encompassed both the combination chemotherapy phase and potential subsequent maintenance. As of the data cutoff (August 30, 2025), five patients (14.3%) remained on zanubrutinib maintenance therapy. Among the 30 patients who discontinued treatment, the primary reasons were disease progression (*n* = 19, 54.3% of the total cohort) and death (*n* = 4, 11.4%).

The best observed overall response rate and complete response rate during treatment were 68.6% (95% CI, 50.7–83.2) and 54.3% (95% CI, 36.6–71.2), respectively. At interim assessment, the ORR was 68.6% (24/35), including 19 complete responses (54.3%) and 5 partial responses (14.3%); 11 patients (31.4%) had progressive disease. After completion of induction therapy, the final ORR was 45.7% (16/35; 95% CI, 28.8–63.4), and the CR rate was 42.9% (15/35; 95% CI, 26.3–60.6). Overall, 19 patients (54.3%) experienced disease progression, including four who initially achieved CR at interim assessment but subsequently relapsed. Among responders, four patients proceeded to autologous stem-cell transplantation and two received subsequent CD19-directed CAR-T therapy.

Subgroup analyses were performed to evaluate the association between baseline characteristics and treatment response at the end of induction therapy (Table 1). Pretreatment disease status emerged as a key determinant of response depth. Patients with relapsed disease achieved a significantly higher complete response rate than those with refractory disease (56.5% vs. 16.7%, *p* = 0.034), with a non-significant trend toward a higher ORR (56.5% vs. 25.0%, *p* = 0.152). Other baseline variables, including age, IPI score, Ann Arbor stage, cell of origin, number of prior treatment lines, and salvage regimen, were not significantly associated with response.

**Table 1:** Subgroup analysis of treatment response in 35 patients with relapsed or refractory double-expressor diffuse large B-cell lymphoma [n (%)]

Characteristics and Treatment Selection	CRR	*p* value	ORR	*p* value
Age		0.315		0.505
≥ 60 years	6/18 (33.3)		7/18 (38.9)	
< 60 years	9/17 (52.9)		9/17 (52.9)	
IPI Score		> 0.999		0.738
0–2	8/18 (44.4)		9/18 (50.0)	
3–5	7/17 (41.2)		7/17 (41.2)	
Ann Arbor Stage		> 0.999		> 0.999
I-II	3/8 (37.5)		4/8 (50.0)	
III-IV	12/27 (44.4)		12/27 (44.4)	
Cell of origin (Hans)		0.564		0.582
GCB	2/3 (66.7)		2/3 (66.7)	
Non GCB	13/32 (40.6)		14/32 (43.8)	
Disease status pre-treatment		0.034		0.152
Refractory	2/12 (16.7)		3/12 (25.0)	
Relapsed	13/23 (56.5)		13/23 (56.5)	
Number of prior lines of therapy		> 0.999		> 0.999
1	10/24 (41.7)		11/24 (45.8)	
≥ 2	5/11 (45.5)		5/11 (45.5)	
Treatment Regimen		0.758		0.843
zR-MINE	3/8 (37.5)		3/8 (37.5)	
zR-DHAP	3/8 (37.5)		3/8 (37.5)	
zR-GemOx	3/6 (50.0)		3/6 (50.0)	
zR-GDP	1/2 (50.0)		1/2 (50.0)	
zR-ICE	2/2 (100.0)		2/2 (100.0)	
zR2	3/9 (33.3)		4/9 (44.4)	

**Fig. 3:**
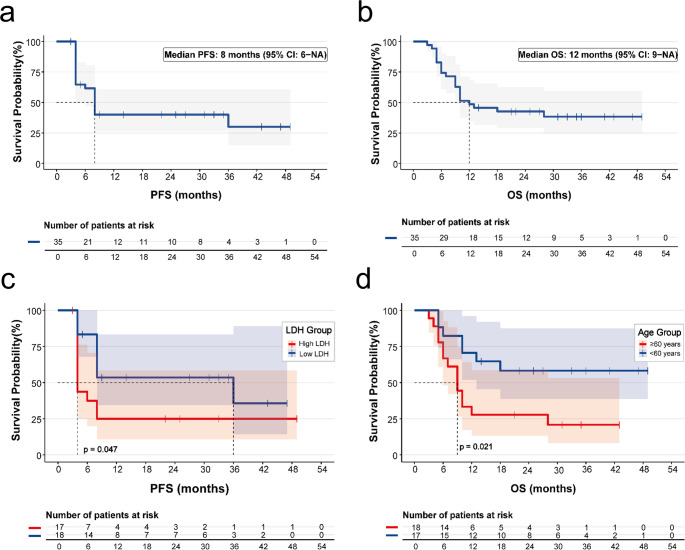
Efficacy outcomes and prognostic analysis of zanubrutinib treatment. (**a**) Kaplan-Meier curve for PFS. (**b**) Kaplan-Meier curve for OS. (**c**) PFS according to baseline LDH level (*p* = 0.047 by log-rank test). (**d**) OS stratified by age group (*p* = 0.021 by log-rank test)

As of August 31, 2025, with a median follow-up of 33 (range, 2–49 months), 23 patients (65.7%) had experienced disease progression or death. One 80-year-old patient with significant cardiovascular risk factors (hypertension, diabetes, and known atherosclerosis) died from multifocal cerebral infarction three months after treatment despite achieving a complete response. Sixteen patients died within 12 months following disease progression, one patient died two years after progression. Additionally, three patients died from SARS-CoV-2 infection; these fatalities occurred during the widespread community outbreak phase in China and while patients were in a chemotherapy interphase, despite their best responses of partial or complete remission. Given the retrospective design of this study, which did not include prospective adjudication of adverse events by an independent committee, a definitive assessment of causality—particularly for the thrombotic, hemorrhagic events and the fatal cerebral infarction—in relation to zanubrutinib treatment cannot be made. Based on the available medical records, these deaths were attributed primarily to disease progression or concurrent infections. Median progression-free survival was 8.0 months (95% CI, 6.0 months to not reached), and median overall survival was 12.0 months (95% CI, 9.0 months to not reached). The 1- and 2-year PFS rate were both 40.1% (95% CI, 26.4–60.8), and the 1- and 2-year OS rates were 48.6% (95% CI, 34.5–68.3) and 42.7% (95% CI, 29.0-62.7), respectively. Furthermore, to assess the robustness of the survival outcomes, a sensitivity analysis censoring deaths not directly attributed to lymphoma progression (three SARS-CoV-2 infections and one cerebral infarction) yielded a median overall survival of 18.0 months (95% CI, 10.0 months to NA) with a stable survival plateau of 43.8% at 36 months (Supplementary Figure S1). Kaplan–Meier survival curves are shown in Fig. 3a and b.

In univariate analysis for overall survival, age ≥ 60 years was associated with inferior OS (HR 2.72, 95%CI, 1.09–6.78; *p* = 0.032), which was confirmed by Kaplan-Meier analysis (log-rank *p* = 0.021; Fig. 3d). Longer duration of response to prior therapy was associated with a trend toward improved OS (HR 0.92, 95%CI, 0.82–1.02; *p* = 0.100). On multivariate analysis, a longer duration of response to prior therapy remained independently associated with improved OS (HR 0.88, 95%CI, 0.77–0.99; *p* = 0.041), whereas age was not independently significant. Sex, Ann Arbor stage, and IPI score were not significantly associated with OS. The detailed results of the univariate and multivariate analyses for OS are summarized in Table 2.

No independent prognostic factors for PFS were identified in multivariate analysis. In univariate analysis, elevated baseline LDH showed a trend toward shorter PFS (HR, 2.08; 95% CI, 0.86-5.00; *p* = 0.103). Consistently, Kaplan-Meier analysis demonstrated significantly inferior PFS in patients with elevated LDH (*p* = 0.047; Fig. 3c).

**Table 2:** Univariate and multivariate analysis for OS

Variable	Univariate Cox		Multivariate Cox	
	HR (95%CI)	*P* value	HR (95%CI)	*P* value
Gender	1.23 (0.52–2.90)	0.640		
Age (< 60; ≥60), years	2.72 (1.09–6.78)	0.032	2.24 (0.82–6.07)	0.114
Ann Arbor stage (I-II; III-IV)	0.87 (0.32–2.38)	0.783		
IPI risk group (0–2; 3–5)	0.91 (0.39–2.14)	0.825		
Cell of origin (Hans) non GCB; GCB	0.55 (0.13–2.40)	0.428		
Bsymptom (yes; no)	1.17 (0.49–2.78)	0.724		
Ecog score (0–1; ≥2)	1.19 (0.46–3.06)	0.726		
Bone marrow involvement (yes; no)	0.69 (0.16–2.97)	0.620		
Serum LDH (normal; rise)	1.91 (0.80–4.57)	0.146	1.48 (0.60–3.62)	0.395
B2MG	0.95 (0.54–1.69)	0.871		
WBC	0.91 (0.77–1.08)	0.293		
HB	1.01 (0.97–1.04)	0.673		
PLT	1.00 (0.99–1.00)	0.064	1.00 (0.99–1.00)	0.054
Ki67 index	1.01 (0.97–1.05)	0.706		
Early treatment (1 line; ≥2 line)	0.95 (0.38–2.37)	0.920		
DOR	0.92 (0.82–1.02)	0.100	0.88 (0.77–0.99)	0.041

*HR* Hazard Ratio, *CI* Confidence Interval, *DOR* Response duration to previous treatment

### Safety

Treatment was generally well tolerated. The most common grade 3–4 hematologic adverse events were neutropenia (42.8%) and thrombocytopenia (14.3%). Febrile neutropenia occurred in 17.1% of patients, and 5.7% required platelet transfusion. The most frequent non-hematologic adverse events were pulmonary infection (34.3%) and fatigue (25.7%), followed by nausea or vomiting (11.4%) and rash (8.6%). Several events of clinical interest occurred: one patient experienced grade 3 hematochezia, which led to a one-week withholding of zanubrutinib and resolved within 3 days with supportive care. Another patient had a grade 3 thrombotic event (lower extremity venous thrombosis) managed with conventional antithrombotic therapy. Additionally, an 80-year-old patient died from multifocal cerebral infarction three months after achieving a complete response. No cases of atrial fibrillation, opportunistic infection, or tumor lysis syndrome were documented in the retrospective records.

## Discussion

Patients with R/R DLBCL carry a poor prognosis, with median OS after conventional salvage therapy approximating just one year [8]. This outlook is particularly guarded for those with the DE subtype—an aggressive subgroup marked by profound chemoresistance and a median OS of less than six months in the R/R setting [9]. The urgent need for more effective therapies is underscored by the observation that even intensive regimens such as DA-EPOCH-R fail to improve outcomes in DE-DLBCL, despite demonstrating benefit in other DLBCL subgroups [10]. In our study, zanubrutinib combined with salvage chemotherapy achieved a median OS of 12.0 months and a CR rate of 42.9%, with the best CR rate reaching 54.3%, representing a clinically meaningful improvement. These findings are consistent with the synergistic activity reported by Wang et al. [11] and further support the rationale for BTK-based combination strategies in this ultra-high-risk setting. Importantly, the sensitivity analysis—which estimated a median OS of 18.0 months and a stable long-term survival plateau—further strengthens the robustness of the survival benefit associated with this regimen.

Real-world data from the REAL-DREND study showed that salvage chemotherapy alone achieved an ORR of 30% and a CR rate of 9%, with a median OS of 5.9 months in R/R DLBCL [12]. Consistent with this, historical data at our center for patients receiving conventional salvage chemotherapy reported a CR rate of only 27.3% [13]. BTK inhibitor monotherapy has demonstrated limited activity; in a phase II study of zanubrutinib monotherapy in R/R non GCB DLBCL, the ORR and CR rate were 29.3% and 17.1%, respectively, with modest PFS and OS outcomes [14]. Patients with the DE phenotype typically respond even more poorly, reinforcing the rationale for mechanism-driven combination strategies. Several early-phase studies have demonstrated the potential of BTK inhibitor–based combinations in R/R DLBCL, including the DE subtype. For example, ibrutinib plus R-ICE achieved an ORR of 90% and a CR rate of 55% [15], while ibrutinib combined with bendamustine-rituximab or lenalidomide-rituximab also showed encouraging activity [16, 17]. Collectively, these studies indicate that BTK inhibitor-based combinations can surmount the limitations of monotherapy. The efficacy observed in our study—ORR 45.7%, CR 42.9%, median OS 12.0 months—compares favorably with these reports, especially considering our cohort was enriched with high-risk DE-DLBCL. This should be interpreted with caution, as our cohort, enriched with relapsed rather than primary refractory patients and those with fewer prior therapies, may represent a population with better underlying prognosis. A recent real-world study by He et al. further supports these results, reporting an ORR of 57.1% and a median PFS of 6.0 months with zanubrutinib-based combinations in transplant-ineligible R/R DLBCL [18]. Together, these data position zanubrutinib-based combinations as a promising therapeutic option.

Mechanistically, the aggressiveness of DE-DLBCL is driven by MYC and BCL-2 overexpression and sustained activation of the B-cell receptor (BCR) signaling pathway. Zanubrutinib, a highly selective BTK inhibitor, effectively suppresses downstream survival and proliferation signals, promoting apoptosis in malignant B cells [19, 20]. This targeted inhibition may sensitize DE-DLBCL cells to chemotherapy, while salvage chemotherapy provides direct cytotoxicity—together contributing to the high CR rate observed in our study. Beyond this synergistic mechanism, appropriate patient selection is critical. Prior studies, including those by He et al., have identified CD79B and MYD88 mutations as strong predictors of response to zanubrutinib-based therapy, with mutation-positive patients achieving markedly higher ORR and longer PFS [18]. Although our study did not include genomic profiling, these findings underscore the importance of incorporating molecular biomarkers into future prospective studies to refine patient selection and optimize therapeutic benefit.

Subgroup analysis identified pretreatment disease status as the primary determinant of response depth. Relapsed patients achieved a significantly higher complete response rate than those with primary refractory disease (56.5% vs. 16.7%, *p* = 0.034). Taken together with the observation that most patients (68.6%) had received only one prior line of therapy, our data raise the possibility that the benefit of this zanubrutinib-based combination might be optimized when introduced earlier in the disease course—for instance, at first relapse and prior to the emergence of broad chemoresistance. The low CR rate in refractory patients underscores the limitations of chemotherapy-based salvage in this setting and highlights the critical need for alternative strategies, such as CD19-directed CAR-T therapy, which has demonstrated superior outcomes in pivotal trials [21]. Other clinical characteristics, including age, IPI score, and regimen type, were not significantly associated with response, and efficacy was similar regardless of treatment intensity.

Multivariate analysis demonstrated that duration of response to prior therapy was an independent predictor of overall survival, suggesting that patients with more durable prior responses derive greater benefit from this combination. A longer prior response may reflect preserved chemosensitivity and less complex clonal evolution, enabling more sustained disease control with combination therapy. These findings further support the potential value of earlier intervention, before the development of broad treatment resistance. In contrast, traditional clinical prognostic factors were not independently associated with survival, raising the possibility that zanubrutinib-based combinations may partially mitigate baseline risk heterogeneity. This hypothesis warrants confirmation in larger, prospective studies.

Our findings, consistent with prior studies in R/R DLBCL [22], showed no significant difference in response between GCB and non-GCB subtypes within our DE-DLBCL cohort. This suggests that cell-of-origin may have limited prognostic relevance in the R/R setting for this subtype, where disease biology is likely dominated by shared pathways such as aberrant BCR signaling and MYC/BCL-2 overexpression. However, the high prevalence of the non-GCB phenotype (91.4%) in our study raises an important consideration regarding the evolving frontline landscape. The phase III POLARIX trial established the superiority of polatuzumab vedotin plus R-CHP (Pola-R-CHP) over R-CHOP-21 in previously untreated ABC-DLBCL [23]. As this regimen becomes standard for eligible patients, its potential to alter the disease biology at relapse may influence the efficacy of subsequent salvage therapies, including zanubrutinib-based combinations—a consideration particularly relevant for younger patients more likely to receive such intensive frontline therapy. Therefore, while targeting core pathogenic pathways like BCR signaling may hold greater clinical relevance than cell-of-origin classification alone, future studies are needed to specifically evaluate the efficacy of BTK inhibitor–based salvage in patients who have relapsed after modern frontline regimens like Pola-R-CHP.

Safety is a critical consideration when evaluating combination regimens. In this study, hematologic toxicities were the most common, with neutropenia (42.8%) and thrombocytopenia (14.3%) occurring at expected frequencies. Given the profound myelosuppression associated with intensive salvage therapy, a robust supportive care strategy was mandated. Central to this strategy was the prophylactic use of pegylated granulocyte colony-stimulating factor alongside a bundle of antimicrobial agents—trimethoprim-sulfamethoxazole, antivirals, and antifungals—to preempt infectious complications, especially in the vulnerable elderly population [24, 25]. This principle of aggressive supportive care to preserve treatment integrity aligns with practices for other potent lymphoma therapies [26].

Although pneumonia was reported in 34.3% of patients, most cases appeared to be associated with disease progression rather than direct treatment toxicity, and no new or unexpected patterns of severe infections were observed. Non‑hematologic toxicities such as nausea, vomiting, and fatigue were consistent with conventional chemotherapy and were generally manageable.

Several events of clinical interest, however, warrant specific discussion. We observed one case of grade 3 hematochezia and one case of grade 3 venous thrombosis. Notably, one elderly patient died from multifocal cerebral infarction after achieving a complete response. Given the retrospective nature of this study, which precluded prospective adjudication of adverse events by an independent committee, a definitive assessment of causality for these specific events in relation to zanubrutinib treatment cannot be made. Considering the established pharmacological profile of BTK inhibitors—which includes a known, albeit potentially reduced with zanubrutinib, association with bleeding risk, platelet dysfunction, and cardiovascular events—these events underscore the necessity for vigilant monitoring [27, 28]. Notably, the observed chemotherapy dose reductions in 28.6% of patients, while reflecting necessary safety management, also indicate an attenuated treatment intensity for a subset of the cohort. The fact that clinically meaningful efficacy was maintained despite this suggests a potential synergistic contribution from zanubrutinib, a hypothesis meriting future prospective evaluation.

The accurate histological diagnosis at relapse or refractory disease is critical, as it may reveal transformation to entities such as double-hit or high grade B cell lymphoma, which would necessitate a different therapeutic approach [29]. While in this retrospective study, not all patients underwent a second biopsy at the time of R/R disease, the clinical imperative for re-assessment is well recognized. Modern interventional radiology techniques, particularly imaging-guided core-needle biopsy (CNB) using ultrasound or CT, have greatly enhanced the safety and feasibility of obtaining adequate tissue for diagnosis in such settings [30, 31]. These minimally invasive procedures allow for timely pathological confirmation and are instrumental in optimizing patient selection for targeted therapies. Future prospective trials evaluating salvage regimens, including those involving zanubrutinib, should ideally incorporate protocols for systematic re-biopsy upon disease progression to accurately characterize the evolving disease biology and guide subsequent therapy.

Beyond these broader diagnostic considerations, several limitations specific to the design of our present study must be acknowledged. First, it was a two-center retrospective study, and the sample size was relatively small, which may introduce selection bias. Second, the use of heterogeneous salvage chemotherapy regimens—reflecting real-world practice—may have contributed to variability in treatment outcomes. Third, the lack of molecular profiling limited our ability to elucidate the mechanistic basis of synergy and to identify predictive biomarkers. Finally, although CAR-T cell therapy is an established option for R/R LBCL, the retrospective design precluded direct comparison with this modality. Future prospective, controlled studies are needed to validate our findings, refine patient selection, and determine the optimal timing and sequencing of zanubrutinib-based therapy in relation to CAR-T and other emerging treatments. Additionally, our reliance on intensive imaging protocols (serial PET/CT and q3-month CT scans) for response assessment and follow-up, while methodologically rigorous, highlights an opportunity to optimize long-term surveillance in clinical practice. Transitioning to less aggressive, patient-friendly strategies—such as ultrasonography-based monitoring for patients in remission—could reduce resource utilization and patient burden without compromising relapse detection, as supported by evidence in other lymphoma settings [32]. Furthermore, our study did not address the financial toxicity associated with this combination. The median treatment duration of 8 months underscores that for responding patients, the long-term cost of targeted therapy plus chemotherapy poses a substantive burden for healthcare systems, warranting future health-economic evaluations.

In conclusion, this retrospective proof of concept study provides preliminary evidence that zanubrutinib combined with salvage chemotherapy exhibits clinically meaningful activity with manageable toxicity in patients with R/R DE-DLBCL. These encouraging results justify and inform the design of future prospective, multicenter randomized trials to definitively establish the efficacy of this combination approach in this high-risk population.

## Supplementary Information

Below is the link to the electronic supplementary material.

Supplementary figure 1 (PNG 236 kb)

Supplementary Material 1

## Data Availability

The study was implemented in accordance with local regulations, the principles of the Declaration of Helsinki and the International Conference on Harmonisation Good Clinical Practice guidelines. The study protocol and all amendments were approved by institutional review boards and independent ethics committees. All patients provided written informed consent.
