# Supplementary figures and images for: Real-world effectiveness of zanubrutinib plus salvage chemotherapy in relapsed or refractory double-expressor DLBCL
Source: Ann Hematol. 2026 Apr 15;105(5):247. doi: 10.1007/s00277-026-06974-6 (PMC13079505; doi:10.1007/s00277-026-06974-6)

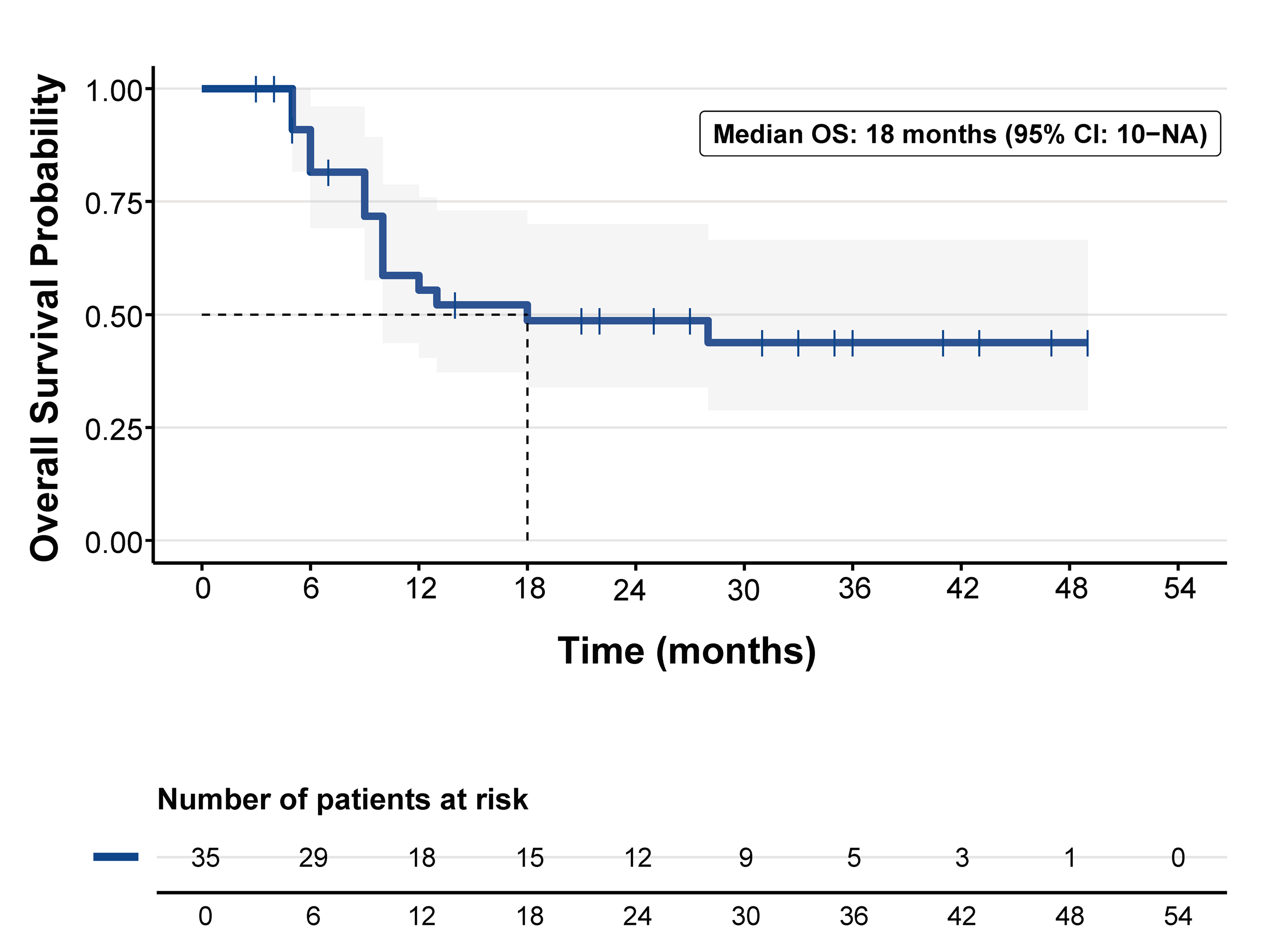

Supplement: Supplementary file 1 — (PNG 236 kb) [file 277_2026_6974_Fig4_ESM.png]

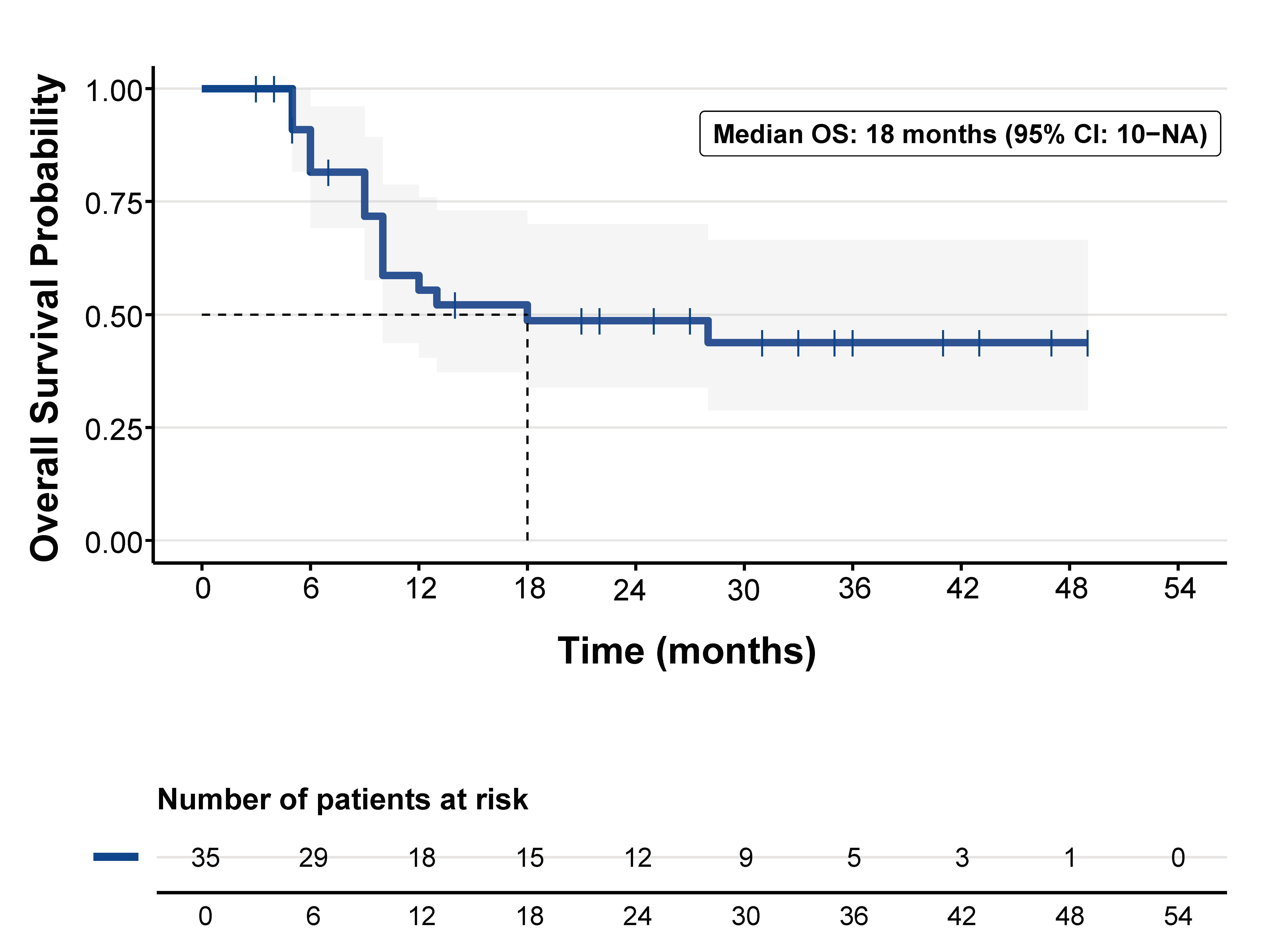

Supplement: Supplementary file 2 — Supplementary Material 1 [file 277_2026_6974_MOESM1_ESM.tif]
